# Effects of tricyclic antidepressants, selective serotonin reuptake inhibitors, and selective serotonin-norepinephrine reuptake inhibitors on the ocular surface

**DOI:** 10.5935/0004-2749.20230068

**Published:** 2023

**Authors:** Ayna Sariyeva Ismayilov, Guler Celikel

**Affiliations:** 1 Department of Ophthalmology, Boyabat 75th Year State Hospital, Sinop, Turkey.; 2 Department of Psychiatry, Boyabat 75th Year State Hospital, Sinop, Turkey.

**Keywords:** Serotonin reuptake inhibitors, Antidepressive agents, tricyclic, Serotonin, Depression, Chronic pain, Dry eye syndromes, Norepinephrine, Surveys and questionnaires, Anxiety, Pharmaceutical preparations, Inibidores de recaptação de serotonina, Antide­pressivos tricíclicos, Serotonina, Depressão, Dor crônica, Síndromes do olho seco, Norepinefrina, Inquéritos e questionários, Ansiedade, Preparações farmacêuticas

## Abstract

**Purpose:**

This study aimed to investigate the effects of tricyclic antidepressants, selective serotonin reuptake inhibitors, and selective serotonin noradrenaline reuptake inhibitors on the ocular surface.

**Methods:**

The study included 330 eyes of 165 patients using antidepressants and 202 eyes of 101 controls. Tear fluid breakup time, Schirmer I test, and Ocular Surface Disease Index (OSDI) questionnaire were administered. Beck Depression Inventory and Beck Anxiety Inventory were applied to record drug use, dosages, psychiatric disease duration, and remission time.

**Results:**

Mean tear fluid breakup time was 14.29 ± 4.81 (4-26) sec, and Schirmer I test value was 16.05 ± 5.89 (2-28) mm in study group. Tear fluid breakup time was 18.16 ± 2.12 (15-24) sec and Schirmer I test value was 16.64 ± 2.31 (15-24) mm in control group (p<0.001 and p=0.005, respectively). In study group, 38.18% (n=63) of patients had dry eye, and 17% (n=18) of patients in control group had dry eye (p<0.001). The mean OSDI score was 82.56 ± 16.21 (66-100) in the tricyclic antidepressants Group, 60.02 ± 29.18 (10-100) in the serotonin reuptake inhibitors Group, and 22.30 ± 20.87 (0-75) in the serotonin-noradrenaline reuptake inhibitors Group (p<0.001). Mean tear fluid breakup time was 14.36 ± 3.35 (10-20) sec in tricyclic antidepressants Group, 13.94 ± 5.81 (4-26) sec in the serotonin reuptake inhibitors Group, and 14.93 ± 4.20 (6-20) sec in serotonin-noradrenaline reuptake inhibitors Group (p=0.730). The mean Schirmer I test value was 9.90 ± 7.22 (2-30) mm in tricyclic antidepressants Group, 15.55 ± 5.15 (2-25) mm in serotonin reuptake inhibitors Group and 17.71 ± 4.21 (10-30) mm in serotonin-noradrenaline reuptake inhibitors Group (p<0.001). There was no statistically significant difference between OSDI score, tear fluid breakup time, and Schirmer I test values in serotonin reuptake inhibitors and serotonin-no­radrenaline reuptake inhibitors subgroups.

**Conclusions:**

Dry eye is common in antidepressant users, but considering the ocular surface, serotonin-noradrenaline reuptake inhibitors may be more reliable than other antidepressants. Patients using serotonin-noradrenaline reuptake inhibitors have lower OSDI scores. Serotonin-noradrenaline reuptake inhibitors, which are useful in chronic pain syndromes, may also have a corrective effect on dry eye symptoms.

## Abstract (pt)

**Objetivo:**

O objetivo deste estudo é investigar os efeitos dos antidepressivos tricíclicos, dos inibidores da recaptação da serotonina e dos inibidores da recaptação da serotonina e noradrenalina na superfície ocular. Métodos: Foram incluídos no estudo 330 olhos de 165 pacientes em uso de antidepressivos e 202 olhos de 101 controles. Foi medido o tempo de ruptura do fluido lacrimal e foram administrados o teste de Schirmer I e o questionário Ocular Surface Disease Index (OSDI). Os Inventários de Depressão e de Ansiedade de Beck foram aplicados ao uso dos medicamentos e foram registrados as dosagens, a duração da doença psiquiátrica e o tempo de remissão.

**Resultados:**

No grupo de estudo, o tempo médio de ruptura do fluido lacrimal foi de 14,29 ± 4,81 segundos (intervalo de 4-26 segundos) e o valor médio do teste de Schirmer I foi de 16,05 ± 5,89 mm (intervalo de 2-28 mm). No grupo controle. o tempo médio de rompimento do fluido lacrimal foi de 18,16 ± 2,12 segundos (intervalo de 15-24 segundos) e o valor do teste de Schirmer I foi de 16,64 ± 2,31 mm (intervalo de 15-24 mm), com p<0,001 e p=0,005, respectivamente. No grupo de estudo, 38,18% (n=63) dos pacientes tinham olho seco, enquanto no grupo controle 17% (n=18) tinham olho seco (p<0,001). O escore médio no OSDI foi de 82,56 ± 16,21 (intervalo 66-100) no grupo dos antidepressivos tricíclicos, 60,02 ± 29,18 (10-100) no grupo dos inibidores da recaptação da serotonina e 22,30 ± 20,87 (0-75) no grupo dos inibidores da recaptação da serotonina e noradrenalina (p<0,001). O tempo médio de rompimento do fluido lacrimal foi de 14,36 ± 3,35 segundos (intervalo de 10-20 segundos) no grupo dos antidepressivos tricíclicos, 13,94 ± 5,81 segundos (intervalo de 4-26 segundos) no grupo dos inibidores da recaptação de serotonina e 14,93 ± 4,20 segundos (intervalo de 6-20 segundos) no grupo dos inibidores da recaptação de serotonina e noradrenalina (p=0,730). O valor médio do teste de Schirmer I foi de 9,90 ± 7,22 mm (intervalo de 2-30 mm) no grupo dos antidepressivos tricíclicos, 15,55 ± 5,15 mm (intervalo de 2-25 mm) no grupo dos inibidores da recaptação da serotonina e 17,71 ± 4,21 mm (intervalo de 10-30 mm) no grupo dos inibidores da recaptação da serotonina e noradrenalina (p<0,001). Não houve diferença estatisticamente significativa no escore OSDI, no tempo de ruptura do fluido lacrimal e nos valores do teste de Schirmer I entre os subgrupos de pacientes em uso de inibidores da recaptação de serotonina e de inibidores da recaptação de serotonina e noradrenalina. **Conclusões:** Olho seco é uma queixa comum em usuários de antidepressivos, mas no que diz respeito à superfície ocular, inibidores da recaptação de serotonina e noradrenalina podem ser mais confiáveis que outros antidepressivos. Pacientes em uso de inibidores da recaptação de serotonina e noradrenalina têm escores menores no questionário OSDI. Os inibidores da recaptação da serotonina e noradrenalina, úteis nas síndromes de dor crônica, também podem ter um efeito corretivo nos sintomas de olho seco.

## INTRODUCTION

Antidepressants use is increasing every day. These drugs are used to treat not only depression but also anxiety disorders, obsessive-compulsive disorders, chronic pain, feeding and eating disorders, somatic symptom and related disorders, and post-traumatic stress disorder. The prescription number increased by an average of 10% per year between 1998 and 2010^(1)^. About half of antidepressants users (about 3.5 million people in England) have been on medication for more than 2 years^(2)^. In the USA, almost 8% of the population aged over 12 years used antidepressants in 1999-2002^(3)^.

Since the eye has a high metabolic rate and rich blood supply, drug side effects are common. Many side effects of psychiatric drugs have been reported^(4)^. In the early period of tricyclic antidepressants (TCAs) use, mydriasis, cycloplegia, and dry eye may occur due to their anticholinergic effect^(4,5)^. Selective serotonin reuptake inhibitors (SSRIs) have been shown to cause such side effects as mydriasis, increased intraocular pressure, and sertraline-induced maculopathy^(6-8)^. Moreover, bilateral acute angle-closure glaucoma attack due to duloxetine, a serotonin noradrenaline reuptake inhibitor (SNRI), has been described in the literature^(9)^.

Dry eye (DE) occurs due to insufficient amount of tears, causing damage to the ocular surface or tear film layer irregularity due to excessive tear evaporation and is associated with ocular disturbance symptoms^(10,11)^. DE prevalence and incidence are increasing with contact lenses, drugs, and electronic device use in the elderly population. It is known that both psychiatric diseases^(12)^ and antidepressants cause DE. Therefore, in our study, patients who were in remission and using antidepressants for at least 8 weeks were examined. The effects of TCAs, SSRIs, and SNRIs on the ocular surface were compared.

## METHODS

A total of 330 eyes of 165 patients using antidepressants for psychiatric diseases who underwent a psychiatric and ophthalmic examination in Boyabat State Hospital between July 2020 and February 2021 and 202 eyes of 101 controls were evaluated in this study. Among the patients who applied to the psychiatry outpatient clinic, whose psychiatric disease diagnosis was confirmed by a review according to the Diagnostic and Statistical Manuel of Mental Disorders, Fifth (DSM-5), and whose treatment was ongoing were included in the study. Additionally, those who met study inclusion and exclusion criteria, agreed to participate, and were in remission during their follow-up for the last 2 months were included in the study. To understand the effects of drugs, and not diseases, on the ocular surface, patients in remission were included in the study.

In antidepressant group (n=165), 30 patients used TCAs (all amitriptyline), 90 patients used SSRIs (subgroups: 29 patients used paroxetine, 29 patients used escitalopram, and 32 patients used sertraline), and 45 patients used SNRIs (subgroups: 25 patients used venlafaxine and 20 patients used duloxetine).

Beck Depression Inventory (BDI) and Beck Anxiety Inventory (BAI) were applied to evaluate for complete recovery in the last 2 months during patients’ clinical follow-ups. Tear fluid break up time (TBUT), Schirmer I test, and Ocular Surface Disease Index (OSDI) questionnaire were administered by an ophthalmologist. This study was conducted in accordance with Declaration of Helsinki. The study was approved by Samsun Ondokuz Mayıs University Local Ethics Committee (ethics committee number: 2021000173-1/2021/173). Furthermore, all participants provided informed consent for their participation in the study.

Patients with glaucoma, those with a history of contact lens use, retinal or refractive surgery, collagen vascular disease, sarcoidosis, renal failure, graft-versus-host disease, thyroid disorder, those aged younger than 18 years, and those having a psychiatric co-diagnosis according to DSM-5 during the evaluation were excluded from the study.

### Beck depression inventory (BDI)

This scale was developed by Beck and associates in 1961 and then revised by Beck in 1979. BDI is a self-ad­ministered questionnaire to screen and assess depression severity in adolescents and adults. Twenty-one scale items assess depression intensity in diagnosed patients and detect possible depression in the normal population. Each item comprises a list of four statements arranged in increasing severity about a particular depression symptom. Most items on BDI are rated on a 4-point scale ranging from 0 to 3. Mild depression is indicated by a score of 11-17, moderate depression is indicated by a score of 18-23, and severe depression is indicated by a score of 24 or higher. The 1979 version of BDI was adapted into Turkish by Hisli in 1988^(13)^.

### Beck anxiety inventory (BAI)

This 21-item scale measures self-reported anxiety severity in adults and adolescents. Descriptive statements of anxiety symptoms are rated on a 4-point scale ranging from 0 to 3 as “not at all” (0); “mildly; it did not bother me much” (1); “moderately; it was very unpleasant, but I could stand it” (2); and “severely; I could barely stand it” (3). The total BAI score is 21-symptom ratings sum, with the maximum possible score of 63 points. According to the 1993 revision of BAI manual, total scores of 0-7 reflect “a minimal level of anxiety”, 8-15 reflect “mild anxiety”, 16-25 reflect “moderate anxiety”, and 26-63 reflect “severe anxiety”. Ulusoy et al. studied BAI validity and confidence in Turkey^(14)^.

Tear fluid break up time (TBUT) (5 µl of fluorescein was instilled and three measurements were made in each eye and averaged) and Schirmer I test (with prior topical anesthesia instilled in the inferior fornix of each eye, Schirmer’s strips were placed and left in the same place for 5 minutes) were performed in the listed order to evaluate DE. Patients with Schirmer I test scores lower than 15 mm or BUT test scores less than 10 seconds were allocated to patient group. The patients filled out a standardized questionnaire regarding DE symptoms (OSDI questionnaire).

### OSDI questionnaire

OSDI is a reliable and validated test to assess DE disease, including 12 questions to assess ocular discomfort, visual symptoms, and environmental factors. OSDI results are documented on a 0-100 scale, with higher scores representing greater disability and more severe disease^(8)^.

### Statistical analysis

Shapiro-Wilk test was used to evaluate variables distribution normality. Continuous variables were presented as mean ± standard deviation and median (minimum-ma­ximum) values. Categorical variables were presented as n (%). Based on the normality test results, Mann-Whitney U test was used to compare two groups, and Kruskal-Wallis test was used to compare more than two groups. Correlation analysis was applied to investigate the relationships between continuous variables, and Spearman’s correlation coefficient was calculated. SPSS (IBM Corp. Released 2012. IBM SPSS Statistics for Windows, Version 21.0. Armonk, NY: IBM Corp.) software was used for the statistical analysis, with p<0.05 indicating statistical significance.

## RESULTS

The study included 330 eyes of 165 patients using antidepressants and 202 eyes of 101 controls. Mean age was 49.09 ± 13.81 (18-79) in study group and 41.90 ± 14.87 (19-76) in control group, without significant difference in the age distribution (p=0.556). There were 136 (82.4%) women in study group and 81 (80.1%) women in control group. There was no significant gender difference between the two groups (p=0.712). Mean TBUT was 14.29 ± 4.81 (4-26) seconds, and Schirmer I test value was 16.05 ± 5.89 (2-28) mm in study group. Mean TBUT was 18.16 ± 2.12 (15-24) seconds, and Schirmer I test value was 16.64 ± 2.31 (15-24) mm in control group. Both values were statistically significantly lower in study group (p<0.001 and p=0.005). In study group, 38.18% (n=63) of patients had DE, and 17% (n=18) of patients had DE in control group (p<0.001) (Table 1).

**Table 1:** Subjects’ Demographics and TBUT and Schirmer I Test Findings

Variable	Study (n=165)	Control (n=101)	p-value
Age	49.09 ± 13.81 (18-79)	41.90 ± 14.87 (19-76)	0.556
Gender (F/M)	136/29	81/20	0.712
TBUT (second)	14.29 ± 4.81 (4-26)	18.16 ± 2.12 (15-24)	**<0.001**
Schirmer (mm)	16.05 ± 5.89 (2-28)	16.64 ± 2.31 (15-24)	**0.005**
Dry eye (yes/no)	63/102 (38.18%/61.82)	18/83 (17.82/82.18)	**<0.001**

Data presented as mean ± standard deviation, median (minimum: maximum). Bold data indicates all values are <0.05.

Independent T test.

TBUT= tear fluid break up time.

Thirty-seven patients (41%) in SSRI group had DE, 18 patients (17%) in control group had DE (p<0.001), and 10 patients (22%) in SNRIs group had DE. There was no statistically significant difference between SNRIs and control groups (p=0.512).

In study group, mean psyshiatric disease duration was 57.53 ± 36 (2-300) months. Mean remission time was 26.64 ± 12 (3-120) months. Fifty-four (20.3%) patients had depression, 97 (36.5%) patients had anxiety disorder, 1 (0.4%) patient had adjustment disorder, 11 (4.1%) patients had somatic symptom and related disorders, and 2 (0.8%) patients had obsessive-compulsive disorder. Among all patients, mean BDI score was 6.19 ± 4 (0-31), and mean BAI score was 6.49 ± 3 (0-33). Thirty (11.3%) patients used TCAs (amitriptyline: mean dosage, 13 mg/d), 90 (33.8%) patients used SSRIs, and 45 (16.9%) patients used SNRIs. In SSRIs group, 29 (10.9%) patients used paroxetine (mean dosage, 22.66 mg/d), 29 (10.9%) patients used escitalopram (mean dosage, 13.96 mg/d), and 32 (12%) patients used sertraline (mean dosage, 91.66 mg/d). In SNRIs group, 25 (9.4%) patients used venlafaxine (mean dosage, 165.66 mg/d) and 20 (7.5%) patients used duloxetine (mean dosage, 44 mg/d) (Table 2).

**Table 2:** Characteristics of population using antidepressants

	n=165
Age (year)	49.09 ± 13.81 (18-79)
Sex (F/M)	136 (81.92%)/29 (18.08%)
The mean duration of psychiatric illness (months)	57.53 ± 36 (2-300)
The mean remission time (months)	26.64 ± 12 (3-120)
Psychiatric illness diagnoses (n/%)	
Depression	54 (20.3)
Anxiety	97 (36.5)
Adjustment disorder	1 (0.4)
Somatic symptom and related disorders	11 (4.1)
Obsessive-compulsive disorder	2 (0.8)
The mean BDI score	6.19 ± 4 (0-31)
The mean BAI score	6.49 ± 3 (0-33)
Used antidepressant (n/%)	
Tricyclic antidepressant (amitriptyline)	30 (11.3)
Serotonin reuptake inhibitor	90 (33.8)
• Paroxetine	29 (10.9)
• Escitalopram	29 (10.9)
• Sertraline	32 (12)
Serotonin and norepinephrine reuptake inhibitor	45 (16.9)
• Venlafaxine	25 (9.4)
• Duloxetine	20 (7.5)
Dry eye (yes/no)	63/102 (38.18%/61.82)

Data are presented as standard deviation, median (minimum: maximum), and n (%).

BDI= Beck Depression Inventory; BAI= Beck Anxiety Score Inventory.

The mean OSDI score was 82.56 ± 16.21 (66-100) in TCAs group, 60.02 ± 29.18 (10-100) in SSRIs group, and 22.30 ± 20.87 (0-75) in SNRIs group (p<0.001). Mean TBUT was 14.36 ± 3.35 (10-20) seconds in TCAs group, 13.94 ± 5.81 (4-26) seconds in SSRIs group, and 14.93 ± 4.20 (6-20) seconds in SNRIs group (p=0.730). The mean Schirmer I test value was 9.90 ± 7.22 (2-30) mm in TCAs group, 15.55 ± 5.15 (2-25) mm in SSRIs group, and 17.71 ± 4.21 (10-30) mm in SNRIs group (p<0.001) (Table 3).

**Table 3:** Mean TBUT, Schirmer I test value, and OSDI score in TCAs, SSRIs, and SNRIs groups

	TCAs group (n=30)	SSRIs group (n=90)	SNRIs group (n=45)	p-value
TBUT (second)	14.36 ± 3.35	13.94 ± 5.81	14.93 ± 4.20	0.730
	(10-20)	(4-26)	(6-20)	
Schirmer Test (mm)	9.90 ± 7.22	15.55 ± 5.15	17.71 ± 4.21	**<0.001**
	(2-30)	(2-25)	(10-30)	
OSDI score	82.56 ± 16.21	60.02 ± 29.18	22.30 ± 20.87	**<0.001**
	(66-100)	(10-100)	(0-75)	

TBUT= tear fluid break up time; OSDI= Ocular Surface Disease Index; TCA= tricyclic antidepressant; SSRI= selective serotonin reuptake inhibitor; SNRI= serotonin-norepinephrine reuptake inhibitor.

Kruskal-Wallis test was used.

The mean OSDI score, Schirmer I test value, and TBUT in TCAs, SSRIs, and SNRIs subgroups are summarized in table 4.

**Table 4:** The mean OSDI score, Schirmer I test value, and TBUT in TCAs, SSRIs, and SNRIs subgroups

	OSDI	Shirmer Test (mm)	TBUT (second)
TCA amitriptyline	82.56 ± 16.21 (66-100)	9.90 ± 7.22 (2-30)	14.36 ± 3.35 (10-20)
SSRI			
Paroxetine	53.64 ± 9.0 (10-100)	14.75 ± 1.35 (2-25)	14.72 ± 1.22 (4-26)
Essitalopram	58.33 ± 7.21 (25-100)	15.51 ± 0.71 (3-22)	13.65 ± 0.88 (5-20)
Sertraline	68.75 ± 8.21 (25-100)	16.31 ± 0.65 (5.25)	13.50 ± 0.95 (5-20)
p-value	0.979	0.996	1
SNRI			
Duloxetine	28.20 ± 12.55 (0-75)	19.50 ± 1.13 (15-30)	15.00 ± 1.7 (7-20)
Venlafaxine	16.40 ± 10.21 (0-25)	16.28 ± 0.54 (10-21)	14.88 ± 0.75 (6-20)
p-value	1	0.218	0.995

TBUT= tear fluid break up time; OSDI= Ocular Surface Disease Index; TCA= tricyclic antidepressant; SSRI= selective serotonin reuptake inhibitor; SNRI= serotonin-norepinephrine reuptake inhibitor.

Kruskal-Wallis test was used.

Mean BDI, BAI, and OSDI scores were 6.13 (0-31), 5.93 (0-33), and 82.56 (66-100) in TCAs group, respectively. There was no correlation between these values (r=0.475, p=0.063 and r=0.130, p=0.630). Mean BDI, BAI, and OSDI scores were 6.02 (0-31), 5.31 (0-33), and 60.02 (10-100) in SSRIs group. There was little correlation between these values (r=0.561 p=0.00 and r=0.474, p=0.004). Mean BDI, BAI, and OSDI scores were 5.24 (0-31), 5.91 (0-31), and 22.30 (0-75) in SNRIs group. There was no correlation between these values (r=-0.52 p=0.483 and r=-0.620 p=0.056).

## DISCUSSION

Prolonged life expectancy, screen exposure, contact lens use, and drug use have increased DE incidence in the modern world. Between 5% and 34% of people suffer from DE^(15,16)^. Two mechanisms are thought to cause DE. One is a decreased tear production from the main lacrimal gland and accessory glands, and the other is rapid tear evaporation secondary to inflammation. DE incidence is higher in both psychiatric patients and patients using antidepressants compared to the general population^(12,17,18)^.

TCAs and, to a lesser extent, SSRIs (paroxetine) reduce lacrimal gland tear production due to their anticholinergic effect, causing DE^(19)^. In this study, Schirmer I test, which evaluated tear production, was lower in TCAs group than in SSRIs and SNRIs groups.

Koçer et al. also reported that SSRIs and SNRIs increased the risk for DE^(20)^. In a study conducted in China, SSRI use alone was an important risk factor for DE^(21)^. In another study on 248 male veterans, SSRI use was asso­ciated with 2.66-times more severe DE symptoms^(22)^. DE was also more frequently found in patients using SSRIs in this study. Although the anticholinergic effect of paroxetine is established, mean TBUT, Schirmer I test value, and OSDI score were similar when paroxetine, sertraline, and escitalopram subgroups were compared. This might have occurred because SSRIs subgroup comprised a smaller number of patients. A study has shown chronic exposure to histamine or 5-hydroxytryptamine (5-HT) induces cytopathologic changes and exocrine dysfunction in *ex vivo* rabbit lacrimal gland acinar cells^(23)^. Chhadva et al. have also reported that patients with DE have higher tear serotonin levels^(24)^. SSRIs block 5-HT transport, which can increase extracellular serotonin concentrations^(25)^. Zhang et al. showed that SSRIs in­duce ocular surface damage and aggravate depression-associated DE via activating the nuclear factor kappa-B (NF-κB) pathway^(26)^. SSRIs can both reduce tear production by damaging the lacrimal gland and cause rapid tear evaporation by initiating inflammation on the ocular surface. Therefore, both Schirmer I test values and TBUT in patients using SSRI were lower than in control group. When SNRIs and control groups were compared, TBUT values in SNRI group were lower, while the Schirmer values were similar. Although Koçer et al.^(20)^ found that SNRIs increased DE incidence, there was no respective difference between control and SSNI groups. SNRIs may be safer for DE than other antidepressants, but this claim requires further support by larger studies.

In the present study, OSDI score was lower in patients using SNRIs than in patients using SSRIs and TCAs. While the inflammatory cascade on the ocular surface persisted for a long time, the central nervous system might have affected and produced central sensitization. When central sensitization develops, the pain occurs independently from peripheral pathology. This can be seen in patients whose DE objective measurements have improved, but symptoms persisted^(27)^. Central sensitization is also seen in diffuse pain syndromes^(28)^. Serotonin and norepinephrine are thought to mediate endogenous analgesia through the descending pain inhibitory pathways in the brain and spinal cord^(29,30)^. SNRIs may reduce pain and improve mood-related symptoms in patients with fibromyalgia. Studies on duloxetine demonstrated its efficacy in the treatment of persistent pain symptoms, depression-associated painful physical symptoms, and diabetic neuropathy-associated pain in patients without depression^(31,32)^. Both duloxetine and venlafaxine might have reduced DE-associated pain in similar mechanisms; therefore, OSDI score in patients using these antidepressants may be lower than in those using other antidepressants.

DE is common in antidepressant users, but regarding the ocular surface, SNRIs may be more reliable than other antidepressants. Patients using SNRIs have lower OSDI scores. SNRIs, which are useful in chronic pain syndromes, may also have an improving effect on DE symptoms.
